# Exploring the Use of Evidence From the Development and Evaluation of an Electronic Health (eHealth) Trial: Case Study

**DOI:** 10.2196/17718

**Published:** 2020-08-28

**Authors:** Monika Jurkeviciute, Henrik Eriksson

**Affiliations:** 1 Centre for Healthcare Improvement Chalmers University of Technology Gothenburg Sweden

**Keywords:** evidence-based practice, evidence use, eHealth, evaluation, evaluation use

## Abstract

**Background:**

Evidence-based practice refers to building clinical decisions on credible research evidence, professional experience, and patient preferences. However, there is a growing concern that evidence in the context of electronic health (eHealth) is not sufficiently used when forming policies and practice of health care. In this context, using evaluation and research evidence in clinical or policy decisions dominates the discourse. However, the use of additional types of evidence, such as professional experience, is underexplored. Moreover, there might be other ways of using evidence than in clinical or policy decisions.

**Objective:**

This study aimed to analyze how different types of evidence (such as evaluation outcomes [including patient preferences], professional experiences, and existing scientific evidence from other research) obtained within the development and evaluation of an eHealth trial are used by diverse stakeholders. An additional aim was to identify barriers to the use of evidence and ways to support its use.

**Methods:**

This study was built on a case of an eHealth trial funded by the European Union. The project included 4 care centers, 2 research and development companies that provided the web-based physical exercise program and an activity monitoring device, and 2 science institutions. The qualitative data collection included 9 semistructured interviews conducted 8 months after the evaluation was concluded. The data analysis concerned (1) activities and decisions that were made based on evidence after the project ended, (2) evidence used for those activities and decisions, (3) in what way the evidence was used, and (4) barriers to the use of evidence.

**Results:**

Evidence generated from eHealth trials can be used by various stakeholders for decisions regarding clinical integration of eHealth solutions, policy making, scientific publishing, research funding applications, eHealth technology, and teaching. Evaluation evidence has less value than professional experiences to local decision making regarding eHealth integration into clinical practice. Professional experiences constitute the evidence that is valuable to the highest variety of activities and decisions in relation to eHealth trials. When using existing scientific evidence related to eHealth trials, it is important to consider contextual relevance, such as location or disease. To support the use of evidence, it is suggested to create possibilities for health care professionals to gain experience, assess a few rather than a large number of variables, and design for shorter iterative cycles of evaluation.

**Conclusions:**

Initiatives to support and standardize evidence-based practice in the context of eHealth should consider the complexities in how the evidence is used in order to achieve better uptake of evidence in practice. However, one should be aware that the assumption of fact-based decision making in organizations is misleading. In order to create better chances that the evidence produced would be used, this should be addressed through the design of eHealth trials.

## Introduction

Evidence-based medicine has taken a central role in health care, aiming to increase the quality of clinical practice. In the medical domain, it is conceptualized as building clinical decisions on credible research evidence, professional experience and judgement, and patient preferences [1-3]. This trend has also risen in the evaluation and implementation of information and communication technologies (ICT) in health care (electronic health [eHealth]) [4,5]. Similar to conventional medicine, decision making in the implementations of eHealth solutions should “rely on explicit evidence derived from rigorous studies on what makes systems clinically acceptable, safe, and effective – not on basic science or experts alone” [6]. Hence, evidence should have utility in these decisions (ie, it should be usable and used). However, there is a growing concern that scientific evidence on whether eHealth works and is safe to use is not sufficiently used when forming health care policies and practice [4,7-11].

Evidence produced by evaluations dominates the discourse related to the evidence-based practice of eHealth implementations [7,12,13]. This emphasis and the strategies to support the use of this type of evidence can be seen through the scholarly discussions and sound methodological base developed in the form of evaluation guidelines, standard measures, and evaluation frameworks [7,13,14]. However, evidence-based practice includes additional types of evidence, such as professional experience and judgement, existing scientific evidence from other research, and patient preferences [1,2,7]. The use of these types of evidence generated through testing and implementing eHealth solutions is underexplored, leading to a lack of supporting strategies.

When the expectation is to use the evaluation evidence in making decisions regarding clinical implementations of eHealth [5], it refers to instrumental use, which is the direct use of the information in decision making and taking action, in order to change existing practice [15-19]. When evidence is not used instrumentally, it is referred to as a waste of resources and efforts contributing to the phenomenon of “pilotism” (remaining in a pilot state and not taken to integration) [20,21]. However, previous research has identified a number of other ways of evidence use [15,17-19,22,23]. Conceptual use refers to a nondirect use of information and perspectives to enhance understanding. Strategic or symbolic use happens when the evidence is brought up to support or confront an existing idea or decision. To the best of our knowledge, the different ways of evidence use (instrumental, conceptual, or symbolic) in the context of eHealth have not been addressed by previous research. Discussions limited to instrumental use potentially provide a too narrow view of actual evidence use in practice.

Furthermore, the considered users of evidence in the context of eHealth are usually limited to policy makers or health care professionals. However, eHealth is a multi-disciplinary field [14], and there might be more beneficiaries of the evidence. Exploring the types of evidence and the ways different actors use it can be worthwhile to support the uptake of evidence in the context of eHealth.

The purpose of this study was to analyze how different types of evidence (such as evaluation outcomes [including patient preferences], professional experiences obtained within the development and evaluation of an eHealth trial, and existing scientific evidence from other research) are used by diverse stakeholders. An additional aim was to identify barriers to the use of evidence and ways to support its use.

## Methods

### Context

This study was built on a case of a multinational and interdisciplinary European Union–funded project (for anonymity reasons, called “Alpha” in this paper). It was a nonpharmacological eHealth trial aimed at improving quality of life and increasing the independence of elderly with mild cognitive impairment and mild dementia. The Alpha trial introduced a case manager role and an ICT platform that consisted of web-based physical and cognitive exercise programs and an activity monitoring device to be used at home. As such, innovations were introduced on both the service model and technological levels.

The Alpha approach was implemented and tested in 4 countries. The project involved 8 partners: 4 care centers (in the aforementioned countries), 2 research and development companies that provided the eHealth solutions, and 2 science institutions. The trial lasted for 6 months. The evaluation included a number of variables, such as clinical efficacy, quality of life, patient adherence to technology, patient and health care professional’s satisfaction, process effectiveness, and a cost-benefit analysis. Clinical professionals were asked to collect patient data using a number of standardized and custom-made questionnaires, as well as to register some data in the registries in Excel files. The evaluation was performed at the end of the project and was finalized in June 2018. During the evaluation, several project partners were charged analyzing the different variables. As it frequently happens in multinational projects, the evaluation had to overcome several practical circumstances such as ethical approvals and systems integration issues that delayed patient recruitment and the start of the trial. This created a situation in which the trial time had to be shortened for some patients, resulting in a smaller dataset than planned.

### Data Collection

The evidence considered in this study included evaluation results (including patient preferences) and professional experiences from the Alpha trial as well as existing scientific evidence from other research. Data were collected through 9 semistructured interviews with all the partners involved in the Alpha project (4 care centers, 2 research and development companies that provided the eHealth solutions, and 2 science institutions) that were conducted 8 months after the evaluation was concluded. The stakeholders were delimited to the partners of the Alpha project, since they had deep knowledge and experience from the trial and they were the primary candidates to consider using the evidence developed. However, the funding institution did not require the project partners to use evidence from the Alpha project.

The interviewee selection followed the principles of purposive sampling [24] and involved the key members of the Alpha teams in every country (see Table 1). At least one interview per partner was conducted. The interviewees were in a position to either use the evidence directly in making decisions that change practice (clinical, technological, scientific) or to decide whether the evidence is worthy of suggesting or presenting to the decision makers in the organizations. The positions of the interviewees and the industry of their work are presented in Table 1. All the interviews lasted one hour, were conducted via Skype, were recorded, and were transcribed.

**Table 1:** Interview respondents.

Stakeholders		Interviewee occupation
**Care centers**		
	Care center 1	Clinical neuropsychologist
	Care center 2	Quality director
	Care center 2	Senior physician
	Care center 3	Head of eHealth^a^ research
	Care center 4	Project manager
**Research and development companies**		
	Research and development company 1	Coordinator of the eHealth group
	Research and development company 2	Project manager and scientific coordinator
**Science institutions**		
	Science institution 1	Director of the research center
	Science institution 2	Scientific coordinator

^a^eHealth: electronic health.

The interviews followed a guide structured around the components of evidence use [18,19,25], such as evidence users, types of impact, evidence already used (and useful) depending on the agenda of the stakeholder, agenda or purpose, quality of research, methodological credibility, relevance and timing for the organization to use the evidence, presentation of the results, and future plans in relation to the evidence.

### Data Analysis

For every stakeholder, we analyzed the following: (1) the types of activities and decisions that were made after the Alpha project ended; (2) the types of evidence (evaluation results from the trial [including patient preferences], professional experiences, or existing scientific evidence from other research) that were used for those activities and decisions, based on the definitions of evidence [1]; (3) in what way the evidence was used (instrumental, conceptual, symbolic) [15,17-19,22]; and (4) barriers to the use of evidence. Instrumental use of evidence was assumed if the evidence obtained from the trial had a direct impact on practice decisions. Conceptual use of evidence was assumed when the evidence from the trial was used indirectly in ways that impacted the understanding, attitudes, and knowledge of the stakeholders but did not cause a change in practice. Symbolic use of evidence was concluded when the stakeholder had used evidence in confirming previous decisions. If necessary, the data were validated with professionals from the Alpha partners.

The findings were grouped by the types of activities and decisions made by the stakeholders using evidence from Alpha. For each activity or decision, the use of evidence is discussed.

## Results

At 8 months after the Alpha project ended, partners in the project (stakeholders) used evidence in the following decisions and activities: (1) integrating or abandoning the Alpha approach in clinical practice, (2) publishing results from the Alpha study, (3) applying for new research funding, (4) supporting regional policymaking, (5) improving technology, and (6) teaching students and health care professionals. Next, the ways that evidence were used by the stakeholders in every decision and activity are explained.

### Integrating the Alpha Approach Into Clinical Practice

At the time of this study, 2 of the 4 care centers had decided to integrate the Alpha approach into clinical practice (care centers 1 and 2). In these care centers, the decision was mainly informed by the evidence from professional experiences. The health care professionals decided to adopt the Alpha approach based on their experiences with usability and adherence to the technology, as well as on patient satisfaction with the service. At the time of the decision, the evaluation results were not available yet. However, the professionals relied on their experience and the existing scientific evidence from other research that demonstrated that technologies and care models similar to Alpha can be beneficial for the patients targeted. The existing research was quite explicit on the benefits of physical and cognitive exercise (with and without the help of technology) for patients with cognitive impairments.

Specific clinical data are not a reason to not try to implement this model. Existing research can provide us such information. <…> Data related to adherence are enough to be interpreted as a useful model for these patients.

Care center 1, clinical neuropsychologist

The decisions to adopt the Alpha approach in care centers 1 and 2 were facilitated by the fact that resources for implementation were available from regional policies supporting and financing care models like Alpha. Care centers 1 and 2 planned to perform deeper statistical analyses on the clinical outcomes, cost, and savings. If a positive effect was found, the results would be disseminated, which would support their decision to integrate the Alpha approach in practice. Hence, in the case of care centers 1 and 2, professional experiences and existing scientific evidence from other research were used instrumentally (directly in decision making and action), while evaluation evidence was used symbolically (to strengthen the already taken decision).

When the decision was made, we didn’t have any results yet. <...> Our experience and preliminary data showed that this model is quite good. <…> Managers trusted our previous evaluations of similar models and thought that it will be the same. <…> We have to redo the economic evaluation to see how much it actually costs and how much we save.

Care center 2, quality director

Care center 3 planned to use the evaluation results to make a decision regarding adopting the Alpha approach in its clinical practice. The organization planned to present the evaluation results to the board and express a need for an eHealth solution like that tested in the Alpha project. Once approval from the board is obtained, the technology can be purchased and integrated in clinical practice. In this case, using the evaluation results in decision making for practice improvement indicates instrumental use.

Once we demonstrate that the results are OK, we are in a position to escalate it to decision makers, and we are able to incorporate it in our organization.

Care center 3, Head of eHealth research

Care center 4 decided to abandon the Alpha approach. Professional experiences were the primary influence on this decision. The concept was abandoned when the staff realized, over the course of the trial, how many resources the new model requires when applying it within the context of care center 4. It was deemed not the right time for the concept to be adopted in the organization. After the project finished, staff’s experiences in the care process of Alpha were presented to management. In the case of care center 4, professional experiences were used instrumentally (directly in decision making and action).

We didn’t pay so much attention to the results of the evaluation. We looked at what does it mean to work with patients in a situation like that. <…> For management, the descriptive conclusions were more interesting than the analysis.

Care center 4, project manager

### Publishing Results of the Alpha Project

Publishing the results from the Alpha project in scientific outlets was initiated by almost all the partners of Alpha (except care center 4). The care centers used the clinical outcomes, quality of life, patient and employee satisfaction, and cost data in the scientific publications. The research and development companies used the adherence data and the feedback from patients and health care professionals related to their specific technology. Publishing the evaluation results helped these companies strengthen their image by demonstrating a case of the technology application in a real setting. In addition to the evaluation results, the partners used the existing scientific evidence from other research to build a case for research.

Since scientific publishing did not directly relate to decision making in practice, such use of evaluation evidence and existing evidence from other research was deemed conceptual.

### Applying for New Research Funding

Most of the partners (care centers 1, 2, and 3; science institutions; and research and development companies) used Alpha evaluation results and professional experiences when applying for further research funding. Evaluation outcomes, experiences, and lessons learned in Alpha provided the basis for the case and allowed ideas to be built that could be applied in the next project. In such a case, Alpha evaluation results and professional experiences did not change local practices but increased the knowledge and understanding of the partners that subsequently helped to develop better research ideas and improve the design of the future studies. Hence, such use of evaluation evidence and professional experiences was deemed conceptual.

### Supporting Regional Policy

Science institutions 1 and 2 and care centers 1, 2, and 3 presented Alpha results in regional policymaking activities as a concrete local example of an eHealth-supported care model within their regions. For this purpose, science institution 2 used the managerial and economic evidence from the evaluation of Alpha to demonstrate the possible impact of the eHealth solution on the local care facility. In addition, science institutions 1 and 2 used the health care professionals’ feedback and perceptions on working with Alpha in their local contexts to demonstrate local applicability. Since the Alpha case served as an example and was not meant to make decisions based on its results, such use of evaluation evidence and professional experiences in policymaking was deemed conceptual.

When you discuss a real case here in <region>, these messages are stronger than to discuss cases in <another country> or to say that literature says these things are useful.

Science institution 2, scientific coordinator

### Improving eHealth Technology

The evaluation results of Alpha helped research and development company 2 in making decisions to improve its technology. The company focused on the patients’ and health care professionals’ feedback and preferences in relation to its technology and initiated actions to improve it. Since the evaluation evidence was used directly for decisions and action, we classified such use of evidence as instrumental.

### Teaching Students and Health Care Professionals

Professional experiences with Alpha were used by science institution 1 in teaching students and health care professionals. Science institution 1 relied on the professionals’ feedback and perceptions on working with Alpha in their local contexts, as it demonstrates local applicability. Since the Alpha case served as an example and decisions were not meant to be made based on its results, such use of professional experiences in teaching was deemed conceptual.

We used the experience with the care models as an example in our courses. <…> We use it for practitioners as a subject to discuss and reflect upon.

Science institution 1, director of the research center

Table 2 describes situations of using the evidence 8 months after the project ended.

**Table 2:** Evidence use by different stakeholders.

Decisions taken and activities		Use of different types of evidence in making the decision or performing the activity		
		Alpha evaluation results	Professional experience with Alpha	Existing scientific evidence from other research
**Care center 1**				
	Adopt Alpha approach	Symbolic	Instrumental	Instrumental
	Publish results	Conceptual	No use observed	Conceptual
	Support regional policy	No use observed	Conceptual	No use observed
**Care center 2**				
	Adopt Alpha approach	Symbolic	Instrumental	Instrumental
	Publish results	Conceptual	No use observed	Conceptual
	Apply for research funding	Conceptual	Conceptual	No use observed
	Support regional policy	No use observed	Conceptual	No use observed
**Care center 3**				
	Present Alpha approach to decision makers for full implementation	Instrumental (planned)	Instrumental (planned)	Instrumental (planned)
	Publish results	Conceptual	No use observed	No use observed
	Apply for research funding	Conceptual	Conceptual	No use observed
	Support regional policy	No use observed	Conceptual	No use observed
**Care center 4**				
	Abandon Alpha approach	No use observed	Instrumental	No use observed
**Science institution 1**				
	Publish results	Conceptual	No use observed	Conceptual
	Teach students and health care professionals	No use observed	Conceptual	No use observed
	Support regional policy	No use observed	Conceptual	No use observed
	Apply for research funding	Conceptual	Conceptual	No use observed
**Science institution 2**				
	Support regional policy	Conceptual	Conceptual	No use observed
	Apply for research funding	Conceptual	Conceptual	No use observed
	Publish results	Conceptual	Conceptual	Conceptual
**Research and development company 1**				
	Publish results	Conceptual	No use observed	Conceptual
	Apply for research funding	Conceptual	Conceptual	No use observed
**Research and development company 2**				
	Improve technology	Instrumental	Instrumental	No use observed
	Publish results	Conceptual	No use observed	Conceptual

### Barriers to the Use of Evidence From the Alpha Trial

The first barrier to the use of evidence was related to the number of variables included in the Alpha evaluation. In most of the project locations, the scope of evaluation was deemed too extensive (it included several variables related to clinical efficacy, quality of life, patient adherence to technology, patient and health care professionals’ satisfaction, a number of variables to assess process effectiveness, and a cost-benefit analysis). The interviewees indicated that the time needed to collect this amount of data for every patient was too long. The clinicians had to fit the data collection into their routine work during meetings with the patients. Consequently, the clinicians were making choices about which data to collect at a particular time. Such trade-offs between data collection for the project and time spent with a patient affected the completeness of data collected and consequently the quality of evidence produced in the evaluation.

When you want to monitor a lot of variables, it is directly related to the time you need to spend with the patients. <…> The target should be to optimize how we collect the variables and information using not that much time.

Care center 3, Head of eHealth research

The second barrier to the use of evidence was related to the Alpha evaluation design when comparing before-after situations. Some of the interviewees with a clinical background disagreed that eHealth integration decisions can be purely based on hard facts and not on evidence from the evaluation when making such a decision. According to these respondents, novel eHealth-supported care models tested during trials are complex dynamic systems within the local context that vary, have differences in culture, and are affected by social interaction. Since these care models cannot be assumed as stable controlled systems, the assumption of stability in the traditional before-after measurement design of an eHealth trial provides less valuable information for eHealth integration decisions to improve practice. Additionally, such an evaluation design comparing before-after situations does not maximize the potential to enhance local learning. The interviewees suggested that people’s experiences with an eHealth solution and process measures, both captured continuously, could lead to iterative adaptation and adjustment between the eHealth solution and the context. Such iterative evaluation would provide higher value in these eHealth integration decisions to improve practice.

If we own the evaluation, we would take repeated measurements for improvement efforts and enhanced learning, rather than traditional approaches.

Care center 2, quality director

## Discussion

### Principal Findings

In this study, we analyzed how different types of evidence, generated through the development and evaluation of an eHealth trial, are used by diverse stakeholders. This work demonstrated that evidence from eHealth trials is used in more ways than decision making regarding clinical integration or policymaking. In addition, different stakeholders can use the evidence for scientific publishing and dissemination, eHealth technology improvement, research funding applications, and teaching.

We found that professional experiences seem to have more influence over decisions regarding eHealth integration into clinical practice than formal evaluations and research. Learning whether and how an eHealth solution could fit within the local context provides key information for local decision making. If the design of an eHealth trial fails to create conditions for professionals to gain experiences, it might prevent learning and obtaining evidence that are crucial to increase the success rate of eHealth trials in their post-pilot phase and reduce “pilotism” [5,20]. Moreover, professional experiences from eHealth trials provide evidence that is valuable for the greatest variety of activities that include disseminating knowledge in various formats, policymaking, teaching, and providing feedback for technology improvement. Evaluation evidence might mostly be valuable for scientific publishing. Existing scientific evidence from other research is another type of evidence that can help to make decisions when it comes to integrating eHealth solutions into clinical practice. However, contextual relevance (eg, location, disease) matters.

To support the use of evaluation evidence, one could consider assessing a few, rather than a large number of, variables during an evaluation. It can help ensure the quality of data collected, preventing from making trade-offs between the time required and quality of the data. Additionally, shorter iterative cycles of evaluation could create better possibilities for health care professionals to gain more experience and use it as evidence in decision making regarding integration of eHealth solutions. Professional experiences could enhance evidence when relevant professionals are included and accumulate experience during eHealth trials. Such an approach could increase the degree of learning and chances that the eHealth solution would be integrated into practice and reach sustainability. Existing scientific evidence collected from previously conducted projects or initiatives in the same location as an eHealth solution is implemented can support decisions regarding eHealth integrations better than scientific evidence obtained from other locations. Research evidence produced in other contexts can be problematic for direct translation into making such decisions. However, it has value for scientific dissemination.

### Limitations

The findings of this study were based on a specific innovation research project funded by the European Union. The interviewee sample was delimited to the partners of the project, since they had deep knowledge of the project and its results and were in the favorable position to use the evidence obtained. However, perspectives of the funding agency, industry, or governments could be a valuable avenue for further research. Similarly, evidence produced in other settings and study designs could provide a different view of the use of evidence. Furthermore, the study captured the situation 8 months after the Alpha project was finished. However, the use of evidence might be more extensive in later stages due to the so-called “gestation period” [25].

### Comparison With Prior Work

Previous research on evidence-based practice usually described clinical implementations and policymaking [5,7] as evidence use in the context of eHealth. By focusing on a wider ecosystem of stakeholders than the traditional focus on health care providers and policymakers, our work identifies more uses of evidence, such as scientific publishing and dissemination, eHealth product and service improvement, applying for research funding, and teaching. Furthermore, we analyzed the use of additional types of evidence [1] such as professional experiences in addition to the traditional focus on evidence generated by evaluations and research [7,12,13].

Our study shows that the typical discourse on the instrumental use (in making decisions) of evidence (and lack of it) [5] does not sufficiently reflect the actual use of evidence in the context of eHealth. Evidence also serves the stakeholders conceptually (increasing knowledge and understanding) and symbolically (supporting already taken decisions) [15,17-19,22]. This suggests that evidence created through eHealth trials can have utility beyond decision making in clinical implementations of eHealth solutions or policymaking. Therefore, evidence that is not used instrumentally might not be a waste of resources and effort (the problem of so-called “pilotism”), as frequently judged by scholars [5,20]. Viewing the use of evidence through different users and the ways of using it can reveal the actual ways in which evidence from a trial is used, help commissioning bodies have realistic expectations on the influence trials can have, and help to design better trials.

Our study indicates that experiences in eHealth trials matter more than facts when it comes to how evidence is used for eHealth integration. The reason for this could be that “hard facts” from evaluation are difficult to straightforwardly implement in a complex health care reality [26,27]. Although it is attractive to think about organizations as rationale organisms and systems [5,28], organizations are rather characterized by decisions and actions that are far from rational. Instead, personal incentives, organizational culture, and other more subjective fields come into play when explaining how decisions are made and organizations develop [27,29,30]. Hence, we believe that the designers of eHealth trials could benefit from understanding how learning and continuous improvement are created and how knowledge development occurs in contemporary health care organizations. In other words, these fields could explain why opinions and subjective knowledge are more important than “hard facts” from an evaluation.

We identified a number of strategies to support the use of evidence in practice in the context of eHealth trials. First, we suggest focusing on a smaller number of variables during evaluation, to ensure the quality of collected data. Such a strategy is contradictory to the ever-expanding evaluation frameworks that include a growing number of variables (eg, [31,32]) and arguments that evaluations should aim to capture as wide an array of outcomes as possible [13]. Second, alternative designs to eHealth evaluation have been discussed in prior research (eg, [14,33]). Our study showed that such designs leading to shorter cycles of evaluation and enabling learning, iteration, and forming experiences that can support decisions could be more beneficial in improving the practice of different stakeholders [34-36]. Failure to produce timely results for decision making is among the barriers to the use of evidence identified by previous research [26].

### Conclusions

We conclude that various stakeholders (such as care centers, research and development companies, science institutions) benefit from evidence differently. Therefore, the delimited focus of research on decision making in clinical settings or policymaking does not capture the actual beneficiaries and realities of evidence use. In addition, when making decisions regarding improving practice, stakeholders do not necessarily rely on the factual evidence produced by evaluations. We conclude that, in the context of eHealth, professional experiences seem to have more influence over decisions than formal evaluations and research. Hence, we suggest that scientific and practical discussions around evidence-based practice in the context of eHealth should include all sorts of evidence (evaluation evidence, professional experiences, existing scientific evidence from other research, and patient preferences). Additionally, it could be beneficial to have an in-depth view on how the evidence is used. This could help expand the conventional focus on clinical settings or policymaking and the direct use of evidence produced by research or evaluation when making decisions. Initiatives to support and standardize evidence-based practice in the context of eHealth should take these complexities into consideration to achieve better uptake of evidence in practice.

## Abbreviations

eHealth: electronic health.
ICT: Information and communication technologies
